# Factors associated with inadequate receipt of components and non-use of antenatal care services in India: a regional analysis

**DOI:** 10.1186/s12889-022-14812-3

**Published:** 2023-01-03

**Authors:** Nilu Nagdev, Felix Akpojene Ogbo, Mansi Vijaybhai Dhami, Thierno Diallo, David Lim, Kingsley E. Agho, on behalf of the Global Maternal and Child Health Research collaboration (GloMACH), Osita Ezeh Ezeh, Daarwin Subramanee, Osuagwu Levi Uchechukwu, Kedir Yimam Ahmed, Abukari Ibrahim Issaka, Pramesh R. Ghimire, Blessing Jaka Akombi-Inyang, Pascal Ogeleka, Tanvir Abir, Rose Victor, Deborah Charwe, Abdon Gregory Rwabilimbo

**Affiliations:** 1 School of Health Sciences, Western Sydney University, Campbelltown Campus, Locked Bag 1797, Penrith, NSW 2571 Australia; 2 Translational Health Research Institute, School of Medicine, Western Sydney University, Campbelltown Campus, Narellan Road and Gilchrist Drive, Campbelltown, NSW 2560 Australia; 3 Riverland Academy of Clinical Excellence (RACE), Riverland Mallee Coorong Local Health Network, SA Health | Government of South Australia, Berri, SA 5343 Australia; 4 Patrick Street Family Practice, 8-22 Patrick Street, PO Box 491, Stawell, VIC 3380 Australia; 5 Belmont Hospital, 16 Croudace Bay Road, Belmont, NSW 2280 Australia; 6 Humanitarian & Development Studies, School of Social Sciences, Western Sydney University, Locked Bag 1797, Penrith, NSW 2751 Australia; 7 African Vision Research Institute (AVRI), University of KwaZulu-Natal, Westville Campus, Durban, 3629 South Africa

**Keywords:** Antenatal care, Women, India, Demographic and health survey, Maternal health

## Abstract

**Background:**

Failure to use antenatal care (ANC) and inadequate receipt of components of ANC pose a significant risk for the pregnant woman and the baby. This study aimed to examine a regional analysis of factors associated with receiving no ANC and inadequate receipt of components of ANC services among Indian women.

**Method:**

Information from 173,970 women of reproductive age 15–49 years from the 2019–21 India National Family Health Survey (NFSH-5) was analysed. Logistic regression analyses that adjusted for cluster and survey weights were conducted to assess the socio-demographic and other factors associated with receiving non-use of ANC and inadequate receipt of components of ANC, respectively, in the six regions and 28 states, and 8 union territories in India.

**Results:**

Across regions in India, 7% of women reported no ANC, and the prevalence of inadequate and adequate receipt of components of ANC in all six regions ranged from 67 to 89% and 8% to 24%, respectively. Of all the 36 federated entities, the prevalence of inadequate receipt of ANC components was less than two-thirds in Tamil Nadu, Puducherry, Andaman and the Nicobar Islands, Odisha, and Gujarat. Our analyses revealed that associated factors vary by region, state, and union territories. Women from poor households reported increased odds of receiving no ANC in North, East and North-eastern regions. Women who reported no schooling in South, East and Central regions were associated with increased odds of receiving no ANC. Women from poor households in Himachal Pradesh, Bihar, Uttar Pradesh, Nagaland, Manipur, Uttar Pradesh, and Madhya Pradesh states reported significantly higher odds of inadequate components ANC than women from rich households. The receipt of inadequate components of ANC was significantly higher among women who never read magazines in Delhi, Ladakh, Karnataka, Telangana, Jharkhand, Maharashtra, Uttar Pradesh, Chhattisgarh, Arunachal Pradesh, Manipur, and Mizoram states in India.

**Conclusion:**

A better understanding of the factors associated with and incorporating them into the short- and long-term intervention strategies, including free financial support from the Indian government to encourage pregnant women from lower socioeconomic groups to use health services across all regions, states and union territories.

**Supplementary Information:**

The online version contains supplementary material available at 10.1186/s12889-022-14812-3.

## Background

Globally, 830 women die daily due to pregnancy- and/or childbirth-related complications [1]. Evidence from the Global Burden of Disease, Injuries and Risk Factors (GBD) study in 2015 revealed that 75% of these maternal deaths could be attributed to inadequate use of antenatal care (ANC) services which may lead to pregnancy or childbirth complications, particularly in low- and middle-income countries (LMICs), including South Asian countries [2]. According to the World Health Organization (WHO), adequate receipt of ANC consists of 4 or more ANC visits and the receipt of 7 or more ANC service components [3]. The components of adequate ANC include: confirming the pregnancy date and expected time of delivery; obstetric examination and blood pressure check; urine and blood test; treatment of diseases as required; tetanus toxoid vaccination; iron and folate acid (IFA) supplementation; and health education on self-care, nutrition and sleeping under insecticide-treated bed-nets [3–5].

The global burden of maternal and child morbidity and mortality due to inadequate receipt of ANC services could be reduced with the effective implementation of health education and health promotion strategies in routine ANC visits [3]. India has one of the highest maternal deaths in South Asia, and it lies between 214 and 300 maternal deaths per 100,000 live births. This estimate shows that the country lags well behind the Sustainable Development Goal (SDG-3) of reducing maternal deaths to less than 70 maternal deaths per 100,000 live births by 2030 [1, 6]. In a recent study conducted in South Asian countries, lower maternal education and household wealth index, a lack of family support for women to access ANC services, a lack of quality ANC counselling and an inadequate supply of required nutritional supplements were barriers to adequate receipt of ANC services [7].

In India, few discrete subnational studies have described relevant factors associated with the non-use or limited use of ANC services as well as the non-receipt of adequate ANC services. A study conducted in the eastern region, Bihar showed that only some 40% of pregnant women attended 4 or more ANC visits and received IFA supplements [8]. A separate study conducted in the northern region of Uttar Pradesh indicated that higher-caste women were significantly more likely to utilise ANC services compared to women belonging to the lower castes [9]. Another study conducted in the same region affirmed that higher socioeconomic status was associated with an increased likelihood of receiving most ANC service components [10]. The findings concurred with a study conducted in the four southern Indian states, women from disadvantaged socioeconomic households were significantly less likely to receive quality ANC compared to their counterparts [11]. Using national-level data, studies have elucidated other determinants of ANC service [12–14]; however, the use of national data can mask subnational differences, particularly given the significant disparities in socioeconomic and health service indicators across India [15].

A recent population-based study on utilisation, equity and determinants of full antenatal care in India found that mothers with low education and low income were associated with full ANC utilization [16]. However, this study only adjusted for a few confounding factors because they used the concentration index method, and the examination of receipt of ANC components was not examined by combining both frequency and receipts of ANC. Hence, investigating both the receipt of ANC and the receipt of components of ANC are important factors for reducing maternal and neonatal mortality, particularly in regions, states and Union territories in India.

Therefore, understanding regions, states and union territories factors associated with inadequate receipt of ANC components and non-use of ANC in India is needed to guide initiatives that can give greater priority to improving maternal and child health services in the country. Hence, this study aimed to investigate the factors associated with inadequate receipt of components and non-use of ANC services in India using HFSH-5. The findings from this study will be relevant to policymakers, health practitioners and women’s health advocates in informing policies and programs to improve ANC and to design initiatives to provide appropriate ANC services to Nigerian women.

## Methods

### Data source

The study used the National Family Health Survey (NFSH-5, also called the 2019–21 India Demographic Health Surveys (DHS) dataset. The NFHS-5 is a population-based survey collected by the Indian Ministry of Health, with technical assistance provided by the Inner-City Fund (ICF) International, Maryland, USA. The NFHS-4 collects data on women’s health (e.g., ANC, births, postnatal care and domestic violence information), infant and young children’s health (e.g., feeding practices), as well as socioeconomic characteristics, encompasses a nationwide sample of women between aged 15 and 49 years from 724,115 interviewed, yielding a response rate of 96.9%.

The overall sample was obtained using a two-stage sampling design for rural and urban areas in India. Villages and census enumeration blocks were the primary sampling units (PSU). In the first stage of the sampling, approximately 300 households were selected from the initial rural and urban PSU, these were further divided into segments of 100–150 households. In the NFHS-5, two out of the selected segments were randomly selected for the NFHS-5 survey, using systematic sampling with probability proportional to segment size, where a cluster was either a PSU or a segment of a PSU. In the second stage, 22 households were randomly selected from each rural and urban cluster using a systematic sampling approach [15]. The response rates were over 95% in every Indian state and territory except 94.0% in Madhya Pradesh [15, 17] and 81% in Chandigarh [15]. Detailed information on the survey methodology is noted in the NFHS-5 reports [15].

In our study, we restricted the analyses to the most recent singleton live birth within the five years preceding the NHHS-5 survey to reduce the potential effect of recall bias, and this approach is consistent with past studies [13]. This approach yielded a weighted total of 173,370 samples.

### Outcome variables

This study considered two outcome variables: The frequency of ANC visits, and the second outcome relates to receiving the recommended essential receipts of ANC components during pregnancy. In the second outcome variable, we created a composite score of receipts of ANC components which comprises a simple count of the receipts of ANC components a pregnant mother received in her most recent birth within the five years prior to the survey. The second outcome variable had a minimum value of zero, indicating that a pregnant mother did not receive any ANC service and a maximum value of nine, indicating that the women received services for all the nine receipts of ANC components. The outcome variables for this study were divided into 3 categories: 1) adequate receipt of ANC components, 2) inadequate receipt of ANC components, and, 3) no ANC visit. In our analysis of regions, states and union territories, adequate receipt of ANC components was assigned ‘0’, inadequate receipt of ANC components was categorised as ‘1’, and adequate receipt of ANC components was assigned ‘0’ no ANC was assigned ‘1’. No ANC visit category included infants whose women never attended any ANC visits during pregnancy. Inadequate receipt of ANC components are infants whose women reported less than four ANC visits during pregnancy and used less than nine receipts of ANC components, and adequate receipt of ANC components are infants whose women attended four or more ANC visits during pregnancy and used all the nine receipts of ANC components.

According to the WHO, appropriate components of ANC include: confirming the pregnancy date and expected time of delivery; obstetric examination and blood pressure check; urine and blood test; treatment of diseases if required; tetanus toxoid vaccination; iron and folate supplementation; and health education on self-care, nutrition and sleeping under insecticide-treated bed-nets [3]. In the present study, the nine components of ANC were based on the NFSH-5 surveyed items. These components included: a) told about pregnancy complications, b) had abdomen examined, c) weight measured during pregnancy, d) given IFA supplementation, e) given the drug for intestinal parasites, f) had tetanus injection before birth, g) blood pressure check, h) urine and i) blood test during pregnancy.

### Study variables

**Fig. 1:**
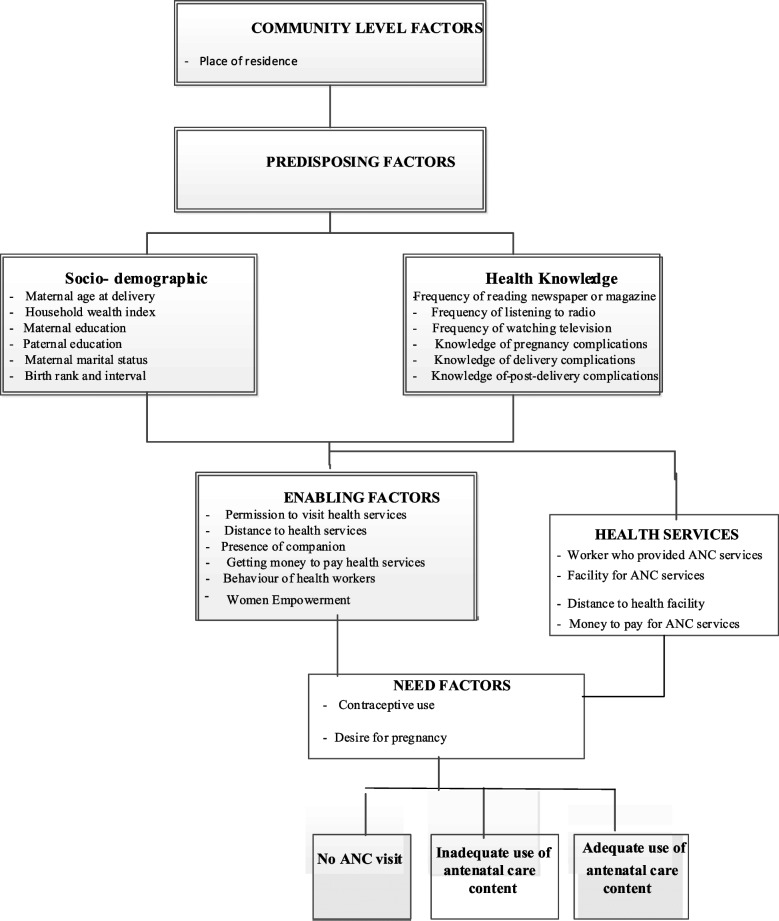
Conceptual framework adapted from Anderson behavioural model

We used the modified Anderson’s behavioural model framework [18] to group the factors potentially associated with inadequate receipt of ANC components (Fig. 1). Twenty-five potential risk factors were identified and categorised into four main groups: 1) community-level factors, 2) predisposing (socio-demographic and health knowledge), 3) enabling including health services and 4) need factors.

The study factors selected were based on Fig. 1 and evidence from previous studies on ANC [14, 19, 20]. The study factors included community and socioeconomic, health knowledge, enabling and need factors. Community factors include the place of residence, and socio-demographic include maternal age at delivery, household wealth index; maternal education; place of delivery; maternal marital status; maternal working status, combined birth rank and interval. The health knowledge factors are frequency of listening to the radio, watching television and reading newspapers, knowledge of delivery complications and knowledge of post-delivery complications. Enabling factors were permission to visit health services, distance to health services, not wanting to go alone to health care, getting money to pay for health services, postnatal checkups and women’s autonomy factors (power over earning, power over household decision-making, wife beaten for refusing sex, attitude to domestic violence). A detailed description of all these study factors is provided in Supplementary Table 1 and the NFHS-5 report [15].

The household wealth index was obtained from a principal components statistical method conducted by the International Institute for Population Sciences (IIPS) and ICF International and was calculated as a score of ownership of about 22 household assets such as transportation devices, and ownership of durable goods and household facilities. The IIPS and ICF International classified the household wealth index into five categories. Each household was assigned to one of these categories: poorest, poorer, middle, rich and richest. These data were re-categorised, where the bottom 40% were referred to as poor households, the next 40% classified as the middle households and the top 20% as rich households to ensure an adequate sample in each category [21, 22] and detailed information regarding the definition and categorisation of potential variables used in the study are provided in Supplementary Table 1.

### Statistical analysis

Data analysis was performed using the survey "svy" commands of Stata version 13.1 (Stata Corp, College Station, TX, USA), which allowed for adjustments for sampling weights. The Taylor series linearization method was used to estimate confidence intervals (CIs) around prevalence estimates in the surveys. First, regional frequency tabulations were conducted to describe the data used in this study, followed by the Taylor series linearization method in the surveys when estimating 95% CIs around the regional prevalence of no ANC, inadequate and adequate receipt of components of ANC. Two logistic regression analyses models that adjusted for clustering and sampling weights were used to identify factors with non-ANC or inadequate receipt of components of ANC for each region, 28 states and 8 union territories, respectively, and detailed information on how the regions were classified has been reported elsewhere [23].

As part of the multivariable logistic analyses, a five-stage model was performed by following a similar conceptual model approach to Andersen’s [24]. In the first modelling stage, community and socioeconomic factors were first entered into the model to assess their associations with the study outcomes. A manually executed backward elimination method was conducted to select factors significantly associated with the outcomes. In the second model, the significant factors in the first stage were added to health knowledge factors, and a manually executed backward elimination procedure followed this. A similar approach was used for enabling health services and need factors in the third, fourth and fifth stages, respectively. To avoid any statistical bias, we double-checked we manually executed the backwards elimination method by using the following procedures: (1) we entered only potential risk factors with *P*-value < 0.20 in the backward elimination process, (2) we tested the backward elimination by also including all variables (all potential risk factors); and (3) we tested and reported any collinearity in the final model. The odds ratio (OR) with 95% CIs were calculated to assess the adjusted risk of independent variables, and those with *P* < 0.05 were retained in the final model.

## Results

### Regional characteristics of the study participants

Table 1 reported the regional characteristics of the study population across all regions in India. More than two-thirds of the women surveyed were aged between 20 and 29 years. As summarised in Table 1, over 50% of the women surveyed who resided in India’s Northern, Southern and Western regions were classified as rich, whilst above 50% of women from the Eastern, Central and North-Eastern regions were poor. Across all the six regions, less than 2% of women were either divorced, separated, or widowed. More than 83% of the women delivered their babies in a health facility, with the highest percentage in the Southern region (98.4%) and the lowest in the North-Eastern region (83.3%). Across all six regions, more than 80% of women reported that their husbands have power over money earned and authority over household purchasing decision-making.

**Table 1:** Characteristics of the study population by regions in India, 2019–21 National Family and Health Survey (*N* = 173,970)

***Characteristic***	**North (*n* = 23,646)**	**South (*n* = 29,541)**	**East (*n* = 44,859)**	**West (*n* = 22,473)**	**Central (*n* = 46,396)**	**Northeastern (*n* = 7055)**
	**n (%)**	**n (%)**	**n (%)**	**n (%)**	**n (%)**	**n (%)**
***Community Level Factor***						
***Type of place of residence***						
Urban	7853 (33.2)	11,891 (40.3)	8221 (18.3)	9675 (43.1)	10,329 (22.3)	1083 (15.3)
Rural	15,793 (66.8)	17,650 (59.8)	36,638 (81.7)	12,798 (57.0)	36,068 (77.7)	5972 (84.7)
***Predisposing factors***						
***Socio-demographic factors***						
**Maternal age at child's birth (Years)**						
< 20	1423 (6.0)	2774 (9.4)	7451 (16.6)	2144 (9.5)	2767 (6.0)	1024 (14.5)
20–29	18,011 (76.2)	21,893 (74.1)	31,383 (70.0)	16,704 (74.3)	35,387 (76.3)	4449 (63.1)
30–39	4062 (17.2)	4728 (16.0)	5674 (12.7)	3504 (15.6)	7793 (16.8)	1482 (21.0)
40 +	150 (0.6)	147 (0.5)	351 (0.8)	121 (0.5)	449 (1.0)	100 (1.4)
**Place of delivery**						
Home	1334 (5.6)	471 (1.6)	7120 (15.9)	1050 (4.7)	6141 (13.2)	1180 (16.7)
Health facility	22,312 (94.4)	29,070 (98.4)	37,739 (84.1)	21,422 (95.3)	40,255 (86.8)	5875 (83.3)
**Wealth Index**						
Rich	13,938 (58.9)	15,939 (54.0)	6807 (15.2)	11,769 (52.4)	14,325 (30.9)	894 (12.7)
Middle	4380 (18.5)	8118 (27.5)	7180 (16.0)	4849 (21.6)	8362 (18.0)	1155 (16.4)
Poor	5328 (22.5)	5484 (18.6)	30,871 (68.8)	5855 (26.1)	23,709 (51.1)	5006 (71.0)
**Employment**						
Did not work	3111 (84.2)	3581 (78.3)	6013 (87.3)	2533 (77.5)	5907 (84.6)	868 (81.1)
Worked	584 (15.8)	994 (21.7)	876 (12.7)	736 (22.5)	1078 (15.4)	203 (19.0)
**Mother's Education**						
Secondary	16,013 (67.7)	25,558 (86.5)	26,525 (59.1)	17,921 (79.7)	28,697 (61.8)	4932 (69.9)
Primary	3075 (13.0)	1775 (6.0)	6094 (13.6)	2190 (9.7)	6146 (13.2)	1104 (15.7)
No Education	4558 (19.3)	2208 (7.5)	12,240 (27.3)	2362 (10.5)	11,553 (24.9)	1018 (14.4)
**Mother's Marital Status**						
Currently married	23,379 (99.0)	29,156 (98.7)	44,364 (99.0)	22,141 (98.7)	45,886 (99.0)	6894 (97.9)
Formerly married	246 (1.0)	374 (1.3)	455 (1.0)	300 (1.3)	459 (1.0)	151 (2.1)
**Combined Birth Rank and Birth Interval**						
2nd/3rd > 2 years	10,279 (43.5)	13,107 (44.4)	19,064 (42.5)	10,011 (44.6)	20,212 (43.6)	3272 (46.4)
1st child	8398 (35.5)	10,877 (36.8)	15,527 (34.6)	8497 (37.8)	13,618 (29.4)	2818 (39.9)
2nd/3rd < = 2 years	3880 (16.4)	5259 (17.8)	6978 (15.6)	3269 (14.5)	8540 (18.4)	523 (7.4)
4th birth > 2 years	752 (3.2)	201 (0.7)	2247 (5.0)	455 (2.0)	2899 (6.2)	349 (4.9)
4th birth < = 2 years	337 (1.4)	97 (0.3)	1043 (2.3)	241 (1.1)	1127 (2.4)	93 (1.3)
***Health knowledge***						
***Frequency of reading magazine or newspaper***						
At least once a week	2649 (11.2)	7160 (24.2)	2695 (6.0)	3768 (16.8)	3652 (7.9)	446 (6.3)
Less than once a week	4709 (19.9)	6929 (23.5)	6995 (15.6)	5345 (23.8)	7426 (16.0)	1093 (15.5)
Never	16,288 (68.9)	15,451 (52.3)	35,169 (78.4)	13,360 (59.5)	35,318 (76.1)	5516 (78.2)
**Frequency of listening radio**						
At least once a week	884 (3.7)	1888 (6.4)	680 (1.5)	841 (3.7)	1438 (3.1)	195 (2.8)
Less than once a week	2396 (10.1)	2889 (9.8)	2674 (5.9)	2099 (9.3)	4324 (9.3)	658 (9.3)
Never	20,366 (86.1)	24,764 (83.8)	41,505 (92.5)	19,533 (86.9)	40,635 (87.6)	6202 (87.9)
**Frequency of watching Television**						
At least once a week	12,978 (54.9)	22,850 (77.4)	16,181 (36.1)	12,788 (56.9)	18,895 (40.7)	2512 (35.6)
Less than once a week	5736 (24.3)	3970 (13.4)	7869 (17.5)	4939 (22.0)	10,293 (22.2)	1819 (25.8)
Never	4933 (20.9)	2722 (9.2)	20,809 (46.4)	4746 (21.1)	17,208 (37.1)	2724 (38.6)
**Told about delivery complications**						
Any complications	17,796 (75.3)	22,159 (75.0)	29,107 (64.9)	17,024 (75.8)	34,662 (74.7)	5109 (72.4)
None	5851 (24.7)	7382 (25.0)	15,752 (35.1)	5449 (24.3)	11,734 (25.3)	1946 (27.6)
**Length of discharge after delivery**						
discharge	733 (3.1)	537 (1.8)	1391 (3.1)	331 (1.5)	1523 (3.3)	138 (2.0)
None	22,913 (96.9)	29,004 (98.2)	43,468 (96.9)	22,141 (98.5)	44,873 (96.7)	6917 (98.0)
***Enabling factors***						
**Getting medical help for self: Getting permission to go**						
No problem	16,103 (68.1)	20,530 (69.5)	26,832 (59.8)	12,971 (57.7)	25,760 (55.5)	4727 (67.0)
Big problem	2846 (12.0)	2942 (10.0)	8424 (18.8)	3153 (14.0)	8947 (19.3)	897 (12.7)
Not a big problem	4698 (19.9)	6069 (20.5)	9603 (21.4)	6349 (28.3)	11,690 (25.2)	1431 (20.3)
**Getting medical help for self: Getting money needed for treatment**						
No problem	13,887 (58.7)	16,632 (56.3)	15,772 (35.2)	12,174 (54.2)	21,661 (46.7)	1908 (27.0)
Big problem	3348 (14.2)	4769 (16.2)	15,242 (34.0)	3160 (14.1)	9754 (21.0)	2618 (37.1)
Not a big problem	6411 (27.1)	8140 (27.6)	13,845 (30.9)	7138 (31.8)	14,982 (32.3)	2529 (35.9)
**Distance to health facilities**						
No problem	11,435 (48.4)	14,414 (48.8)	14,996 (33.4)	10,225 (45.5)	18,137 (39.1)	2107 (29.9)
Not a big problem	7390 (31.3)	9424 (31.9)	15,774 (35.2)	7618 (33.9)	17,348 (37.4)	2780 (39.4)
Big problem	4821 (20.4)	5703 (19.3)	14,089 (31.4)	4630 (20.6)	10,912 (23.5)	2168 (30.7)
**Getting medical health for self not wanting to go alone**						
No problem	12,774 (54)	17,647 (59.7)	19,163 (42.7)	11,396 (50.7)	20,777 (44.8)	3168 (44.9)
Big problem	3761 (15.9)	3532 (12.0)	11,012 (24.6)	3572 (15.9)	8803 (19.0)	1177 (16.7)
Not a big problem	7111 (30.1)	8362 (28.3)	14,685 (32.7)	7505 (33.4)	16,816 (36.2)	2710 (38.4)
**Timing of postnatal check-up**						
0–2 days	1852 (7.8)	2664 (9.0)	3389 (7.6)	1152 (5.1)	4812 (10.4)	260 (3.7)
3–41 days	8981 (38.0)	11,654 (39.5)	16,922 (37.7)	7913 (35.2)	17,793 (38.4)	1963 (27.8)
Don’t know	12,814 (54.2)	15,222 (51.5)	24,548 (54.7)	13,409 (59.7)	23,791 (51.3)	4831 (68.5)
***Health Services***						
**Antenatal care service attendant**						
Health practitioner	20,693 (92.8)	28,405 (97.7)	34,241 (86.1)	20,289 (94.2)	37,359 (91.9)	5967 (91.6)
No one	1285 (5.8)	570 (2.0)	4850 (12.2)	1101 (5.1)	2371 (5.8)	482 (7.4)
Traditional	325 (1.5)	102 (0.4)	682 (1.7)	139 (0.6)	921 (2.3)	67 (1.0)
**Place received antenatal care services**						
Government	7835 (48.0)	11,061 (42.9)	6016 (24.0)	5161 (28.8)	7944 (30.5)	2649 (59.7)
Private	5870 (36.0)	10,582 (41.0)	10,625 (42.3)	6526 (36.4)	9777 (37.5)	735 (16.6)
Home	2609 (16.0)	4150 (16.1)	8455 (33.7)	6266 (34.9)	8337 (32.0)	1051 (23.7)
***Need factors***						
**Contraceptive use**						
Yes	15,408 (65.2)	16,130 (54.6)	27,126 (60.5)	12,104 (53.9)	27,869 (60.1)	4293 (60.9)
No	8238 (34.8)	13,411 (45.4)	17,733 (39.5)	10,368 (46.1)	18,527 (39.9)	2762 (39.1)
**Intention to become pregnant**						
Then	21,587 (91.3)	28,268 (95.7)	39,838 (88.8)	21,279 (94.7)	42,562 (91.7)	6546 (92.8)
Later	1098 (4.6)	703 (2.4)	2623 (5.8)	594 (2.6)	1785 (3.8)	233 (3.3)
No more	961 (4.1)	570 (1.9)	2398 (5.3)	600 (2.7)	2050 (4.4)	276 (3.9)
***Autonomy variables***						
**Power over earnings**						
By husband	21,055 (89.0)	26,383 (89.3)	39,721 (88.6)	20,121 (89.5)	41,361 (89.2)	6238 (88.4)
Woman alone	2591 (11.0)	3158 (10.7)	5138 (11.5)	2352 (10.5)	5035 (10.9)	817 (11.6)
**Power over household decision making**						
By husband	20,502 (86.7)	25,689 (87.0)	38,974 (86.9)	20,121 (89.5)	40,501 (87.3)	6097 (86.4)
Woman alone	3144 (13.3)	3852 (13.0)	5885 (13.1)	2352 (10.5)	5895 (12.7)	958 (13.6)
**Wife beaten for refusing sex**						
Yes	284 (1.2)	720 (2.4)	738 (1.6)	334 (1.5)	754 (1.6)	99 (1.4)
No	23,363 (98.8)	28,820 (97.6)	44,121 (98.4)	22,139 (98.5)	45,642 (98.4)	6956 (98.6)
**Attitudes to domestic violence**						
Yes	828 (3.5)	3108 (10.5)	2109 (4.7)	1031 (4.6)	2334 (5.0)	294 (4.2)
No	22,818 (96.5)	26,433 (89.5)	42,751 (95.3)	21,442 (95.4)	44,062 (95.0)	6761 (95.8)

### Regional prevalence of receipt of components of ANC visits

Table 2 revealed the regional prevalence receipt of components of ANC visits received by Indian women during their last pregnancy. Over 90% of women who lived in the Northern, Southern, North-Eastern and Western regions had their abdomen examined, weight measured, blood pressure checked, and urine and blood test during pregnancy. Over 85% of women received IFA supplementation across all the regions in India.

**Table 2:** Regional prevalence and 95% confidence intervals (CIs) of components of antenatal care received by Indian women during the last pregnancy, NFSH-5

Component	North	South	East	West	Central	Northeastern	National
	% (95%CI)	% (95%CI)	% (95%CI)	% (95%CI)	% (95%CI)	% (95%CI)	% (95%CI)
Told about pregnancy complications	79.6 (78.7,80.4)	76.5 (75.5,77.4)	72.8 (71.8,73.7)	79.7 (78.3,81.0)	78.7 (77.9,79.5)	77.7 (76.5,79.0)	77.1 (76.6,77.4)
During pregnancy, given iron tablets	86.1 (85.5,86.8)	94.4 (93.9,94.8)	85.2 (84.5,85.8)	87.3 (86.3,88.3)	87.0 (86.5,87.5)	91.1 (90.4,91.7)	87.9 (87.6,88.1)
Drugs for intestinal parasites during pregnancy	21.9 (21.1,22.8)	45.5 (44.4,46.6)	28.2 (27.3,29.2)	29.4 (27.9,30.9)	34.1 (33.3,34.9)	14.0 (13.1,14.9)	31.4 (30.9,31.8)
Number of tetanus injections before birth	83.3 (82.6,83.9)	85.6 (84.9,86.2)	84.3 (83.6,84.8)	80.8 (79.6,81.9)	82.9 (82.3,83.4)	86.4 (85.6,87.2)	83.6 (83.3,83.9)
During pregnancy: blood pressure taken	97.9 (97.6,98.1)	99.7 (99.5,99.7)	93.3 (92.9,93.7)	98.6 (98.4,98.9)	94.4 (94.0,94.8)	97.8 (97.3,98.1)	96.2 (96.1,96.4)
During pregnancy: blood sample taken	97.1 (96.8,97.4)	99.6 (99.5,99.7)	89.1 (88.5,89.6)	98.5 (98.2,98.7)	91.9 (91.4,92.3)	94.6 (93.9,95.2)	94.2 (94.0,94.4)
During pregnancy: urine sample taken	95.7 (95.3,96.1)	95.7 (95.3,96.1)	89.3 (88.7,89.8)	98.1 (97.7,98.4)	90.4 (89.8,90.9)	94.3 (93.6,94.8)	93.6 (93.4,93.8)
During pregnancy weighed	98.2 (98.0,98.4)	99.7 (99.7,99.8)	95.1 (94.7,95.4)	99.3 (99.1,99.4)	95.2 (94.8,95.5)	98.4 (98.0,98.7)	97.0 (96.9,97.1)
Abdomen examined	94.2 (93.8,94.7)	98.9 (98.6,99.1)	87.6 (86.9,88.2)	97.7 (97.3,98.0)	89.4 (88.9,89.9)	93.3 (92.6,94.0)	92.5 (92.3,92.7)

The proportion of pregnant women who received intestinal parasites drugs as part of ANC varied from 14% to 45.5%, with the highest rate in the Southern region (45.5% of the study sample) and the North-Eastern region having the lowest reported rate (14%). Nationally, about half of the components of ANC were suboptimal (< 90%) and these included, told about pregnancy complications, IFA and intestinal parasites drugs (see Table 2 for details).

### Regional prevalence of no, inadequate and adequate receipts of components of ANC

**Fig. 2:**
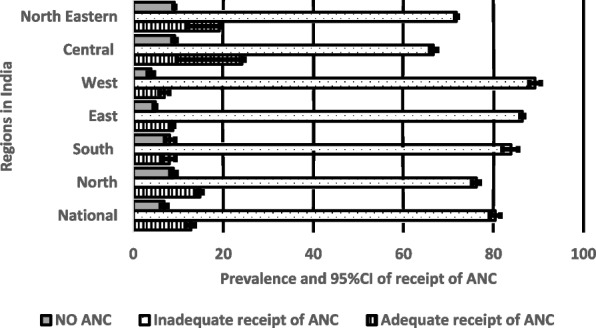
Regional prevalence and 95%CI of no, inadequate and adequate receipt of the components of ANC visits by Regions in India, NFSH-5

Figure 2 shows the regional prevalence and 95% CIs of no ANC, inadequate receipts, and adequate receipts of ANC components by regions in India. The prevalence of no ANC ranged between 5 and 9% in North-Eastern, Central and Eastern regions, with the overall national prevalence of 6.8% (95%CI: 6.6–7.0). The prevalence of no ANC was significantly higher in the Eastern and Central regions and was lower in the West and Eastern regions. Inadequate receipt of ANC components lies between 72 and 89%, the lowest prevalence is in the North-Eastern region, and the highest prevalence is in the Western region, with an overall prevalence of 80.4% (95%CI: 80.1–80.7) across India.

The Central region had a significantly lower prevalence of inadequate receipt of ANC components. In contrast, the prevalence of inadequate receipt of ANC components did not differ statistically in the Eastern and South regions. The Eastern and Western regions reported the highest prevalence of adequate receipt of ANC components, while the Central region reported the lowest (see Fig. 2).

The prevalence of receipt of ANC by the 28 states and 8 Union territories in India was presented in supplementary Fig. 1. The prevalence of no ANC visits was over 1.0% in Nagaland, Manipur, Odisha, Gujarat, Maharashtra, Karnataka, Goa, and Lakshadweep and less than 5.0% in Rajasthan, Uttar Pradesh and Bihar. Of all the 36 federated entities, the prevalence of inadequate receipt of ANC components was less than 7.0% in 5 entities: Tamil Nadu, Puducherry, Andaman and the Nicobar Islands, Odisha and Gujarat. The prevalence of adequate receipt of ANC components was over 30% in Tamil Nadu, Puducherry, Andaman and Nicobar Islands and Odisha (see Supplementary Fig. 1 states for details).

### Factors associated with no ANC

The odds of no ANC visit were significantly higher among women who reported no postnatal checkup after delivery in all five regions except the North-eastern region of India. No ANC visit was significantly higher among women with no education in South, East and Central regions, and no ANC visit was significantly higher among women with limited knowledge of delivery complications in all regions except in the North-eastern region. Women from poor households in in North, East and North-eastern regions significantly reported higher odds of no ANC visit (Table 3).

**Table 3:** Factors associated with no receipts of ANC components

**Variable**	North (*n* = 23,646)	South (*n* = 29,541)	East (*n* = 44,859)	West (*n* = 22,473)	Central (*n* = 46,396)	North Eastern (*n* = 7055)
	AOR (95% CI)	AOR (95% CI)	AOR (95% CI)	AOR (95% CI)	AOR (95% CI)	AOR (95% CI)
***Community Level Factor***						
**Type of residence**						
Urban	1.00	1.00				
Rural	1.27 (1.14,1.4)	1.26 (1.13,1.40)				
**Maternal age at birth**						
< 20 years			1.00	1.00		
20–29 years			0.76 (0.64,0.91)	0.82 (0.68,1.00)		
30–39 years			0.54 (0.43,0.68)	0.75 (0.58,0.97)		
40 + years			0.50 (0.25,1.03)	1.30 (0.70,2.40)		
**Place of delivery**						
Home			1.00	1.00	1.00	1.00
Health facility			0.58 (0.46,0.72)	0.71 (0.55,0.92)	0.63 (0.54,0.74)	1.48 (1.27,1.71)
**Household wealth Index**						
Rich	1.00		1.00			1.00
Middle	1.24 (1.10,1.38)		1.18 (0.99,1.41)			1.21 (0.98,1.50)
Poor	1.26 (1.12,1.42)		1.21 (1.02,1.44)			1.36 (1.12,1.65)
**Mother's education**						
Secondary		1.00	1.00		1.00	
Primary		1.10 (0.92,1.31)	1.02 (0.87,1.21)		1.38 (1.20,1.59)	
No schooling		2.05 (1.70,2.47)	1.30 (1.10,1.52)		1.25 (1.11,1.41)	
**Combined Birth rank and Birth Interval**						
2nd/3rd > 2 years	1.00	1.00	1.00	1.00	1.00	1.00
1st child	1.11 (1.02,1.21)	1.11 (1.02,1.22)	1.27 (1.11,1.44)	1.22 (1.08,1.38)	1.06 (0.95,1.17)	1.01 (0.88,1.15)
2nd/3rd < = 2 years	1.25 (1.11,1.41)	1.53 (1.35,1.73)	2.37 (1.95,2.87)	1.15 (0.98,1.35)	1.28 (1.12,1.46)	1.42 (1.12,1.79)
4th birth > 2 years	1.36 (1.05,1.75)	1.01 (0.57,1.77)	2.22 (1.56,3.18)	1.45 (1.01,2.08)	1.22 (0.98,1.52)	0.91 (0.73,1.14)
4th birth < = 2 years	1.01 (0.70,1.45)	2.46 (0.90,6.77)	2.62 (1.58,4.33)	1.39 (0.91,2.13)	1.33 (0.94,1.86)	0.53 (0.37,0.78)
***Health knowledge***						
**Frequency of listening radio**						
At least once a week	1.00					
Less than once a week	1.16 (0.95,1.43)					
Not at all	1.39 (1.16,1.67)					
**Frequency of reading magazine or newspaper**						
At least once a week	1.00	1.00				1.00
Less than once a week	0.84 (0.73,0.97)	0.82 (0.73,0.94)				1.16 (0.92,1.47)
Not at all	1.00 (0.88,1.14)	0.75 (0.67,0.84)				1.44 (1.16,1.79)
**Frequency of watching Television**						
At least once a week		1.00	1.00		1.00	
Less than once a week		1.20 (1.05,1.37)	1.15 (0.98,1.34)		1.13 (1.00,1.28)	
Not at all		1.29 (1.10,1.52)	1.50 (1.30,1.74)		1.35 (1.20,1.51)	
**Knowledge of delivery complications**						
Yes	1.00	1.00	1.00	1.00	1.00	
No	2.96 (2.55,3.43)	1.69 (1.25,2.28)	4.49 (3.61,5.58)	2.54 (2.15,3.00)	8.81 (6.89,11.25)	
**Post-delivery complications knowledge**						
Yes		1.00				
No		5.58 (4.74,6.56)				
***Enabling factors***						
**Money to pay for health services**						
No problem						
Not a big problem						
Big problem						
**Not wanting to go alone to health care**						
No problem						
Not a big problem						
Big problem						
**Permission to visit health services**						
No problem						
Not a big problem						
Big problem						
**Distance to a health facility**						
No problem		1.00				1.00
Not a big problem		1.21 (1.08,1.37)				1.17 (0.99,1.38)
Big problem		1.34 (1.17,1.53)				1.25 (1.04,1.51)
**Postnatal check-up (PNC)**						
0–2 days	1.00	1.00	1.00	1.00	1.00	
3–41 days	0.78 (0.67,0.90)	0.81 (0.70,0.95)	0.93 (0.76,1.14)	0.85 (0.67,1.08)	0.82 (0.69,0.96)	
No PNC	1.26 (1.08,1.48)	1.52 (1.30,1.78)	1.71 (1.38,2.12)	1.41 (1.10,1.81)	1.21 (1.03,1.43)	
*Autonomy variables*						
**Power over household decision making**						
By Husband alone			1.00			
Alone/joint decision			0.82 (0.69,0.99)			
**Power over earning**						
yes						
no						
**Attitudes to domestic violence**						
Yes						
No						
***Health Services***						
**Contraceptive use**						
Yes	1.00		1.00	1.00	1.00	
No	1.14 (1.04,1.25)		1.17 (1.04,1.32)	1.21 (1.08,1.37)	1.14 (1.03,1.26)	
**Intention to become pregnant**						
Now		1.00		1.00	1.00	
Later		2.30 (1.65,3.21)		1.51 (1.00,2.29)	1.40 (1.04,1.88)	
No more		1.37 (1.00,1.88)		1.67 (1.09,2.55)	1.85 (1.40,2.44)	

If 95% confidence intervals (CI) around AORs that lies between 1.00 indicate not statistically significant

In all regions, the odds of no ANC visit were higher among first time mothers. Women who did not use contraceptives reported higher odds of no ANC in all regions except in the South and North-eastern regions. Similarly, the odds of no ANC visit were significantly higher among women who reported that distance to health facilities was not a big problem in the South and North-Eastern regions. Women who had no intention to become pregnant were significantly more likely to make no ANC visits in the East, West and Central regions.

Women who had never listened to the radio significantly reported higher odds of no ANC visit in the Northern (AOR = 1.99; 95%CI: 1.42 to 2.79) and Southern regions (AOR = 1.41; 95%CI: 1.17 to 1.68) and women who never watched television reported increased odds of receiving no ANC visit in the South (AOR = 1.29; 95%CI: 1.10 to 2.52), East (AOR = 1.50; 95%CI: 1.30 to 1.74) and Central regions (AOR = 1.35; 95%CI: 1.20 to 1.51). Women who lived in rural areas in North and South regions, and their first child reported higher odds of receiving no ANC in all regions except Central and North-eastern regions.

In the state analysis for no ANC, older women (30 +) and women with limited post-delivery complication in Delhi. Women who never watched television and had no PNC in Jammu Kashmir reported higher odds of no ANC visit. Women who delivered their babies in Uttar Pradesh state and women who never watched television in Kerala states in South India were significantly more likely to make no ANC visits. Women from Mizoram in India who did not use contraceptives reported higher odds of No ANC and women from poor households in Arunachal Pradesh state (AOR = 2.75; 95%CI: 1.82 to 4.16) and women from Tripura state with limited knowledge of delivery complication AOR = 1.49; 95%CI: 1.00 to 2.65) were significantly more likely make no ANC visits (see, Tables 1–6 in Supplementary Table 2 for details).

### Factors associated with inadequate receipt of ANC components

As shown in Table 4, the receipt of inadequate components of ANC was significantly lower among women with no schooling in all regions except the West region, and the receipt of inadequate components of ANC was significantly lower among women from poor and middle-income households in the East, West and North-eastern regions. The receipt of inadequate components of ANC was low among women who listened to the radio at least once a week.

**Table 4:** Factor associated with inadequate receipt of ANC

Variable	North (*n* = 23,646)	South (*n* = 29,541)	East (*n* = 44,859)	West (*n* = 22,473)	Central (*n* = 46,396)	North Eastern (*n* = 7055)
	**AOR (95% CI)**	**AOR (95% CI)**	**AOR (95% CI)**	**AOR (95% CI)**	**AOR (95% CI)**	**AOR (95% CI)**
***Community Level Factor***						
**Type of residence**						
Urban	1.00		1.00		1.00	
Rural	1.19 (1.03,1.37)		0.73 (0.59,0.90)		0.64 (0.52,0.80)	
**Maternal age at birth**						
< 20 years		1.00	1.00			
20–29 years		1.30 (1.07,1.59)	1.12 (0.93,1.35)			
30–39 years		1.36 (1.05,1.75)	1.37 (1.08,1.74)			
40 + years		2.71 (1.40,5.26)	1.59 (0.83,3.04)			
**Place of delivery**						
Home	1.00				1.00	1.00
Health facility	0.67 (0.54,0.82)				1.54 (1.11,2.13)	1.76 (1.13,2.73)
**Household wealth Index**						
Rich			1.00	1.00		1.00
Middle			0.84 (0.68,1.04)	0.94 (0.80,1.11)		0.76 (0.57,1)
Poor			0.78 (0.64,0.96)	0.83 (0.70,0.97)		0.72 (0.55,0.95)
**Mother's education**						
Secondary	1.00	1.00	1.00		1.00	1.00
Primary	0.73 (0.60,0.89)	0.80 (0.60,1.06)	0.92 (0.76,1.11)		0.70 (0.52,0.94)	0.68 (0.48,0.98)
No schooling	0.73 (0.62,0.87)	0.63 (0.48,0.82)	0.85 (0.73,1.00)		0.57 (0.43,0.76)	0.54 (0.33,0.86)
**Combined Birth rank and Birth Interval**						
2nd/3rd > 2 years		1.00	1.00		1.00	1.00
1st child		0.85 (0.74,0.97)	0.80 (0.69,0.93)		0.90 (0.74,1.09)	0.94 (0.75,1.18)
2nd/3rd < = 2 years		0.75 (0.62,0.90)	0.57 (0.47,0.70)		0.76 (0.59,0.98)	0.65 (0.43,0.99)
4th birth > 2 years		1.09 (0.49,2.44)	0.76 (0.56,1.02)		0.38 (0.20,0.69)	0.85 (0.5,1.46)
4th birth < = 2 years		0.29 (0.10, 0.84)	0.60 (0.39,0.92)		0.77 (0.35,1.72)	1.24 (0.58,2.65)
***Health knowledge***						
**Frequency of listening radio**						
Almost every day	1.00	1.00	1.00	1.00	1.00	1.00
At least once a week	0.90 (0.68,1.17)	1.42 (1.02,1.97)	0.55 (0.34,0.91)	0.53 (0.33,0.86)	1.84 (1.12,3.02)	0.71 (0.47,1.05)
Less than once a week	0.69 (0.55,0.88)	1.13 (0.86,1.47)	0.57 (0.36,0.89)	0.60 (0.42,0.85)	1.46 (0.94,2.27)	0.42 (0.29,0.61)
**Frequency of reading magazine or newspaper**						
At least once a week						
Less than once a week						
Never						
**Frequency of watching Television**						
At least once a week					1.00	
Less than once a week					0.67 (0.52,0.86)	
Never					0.78 (0.62,0.99)	
**Knowledge of delivery complications**						
Yes	1.00		1.00			
No	1.18 (1.01,1.38)		1.24 (1.08,1.41)			
**Post-delivery complications knowledge**						
Yes						
No						
***Enabling factors***						
**Money to pay for health services**						
No problem						
Not a big problem						
Big problem						
**Not wanting to go alone to health care**						
No problem						
Not a big problem						
Big problem						
**Permission to visit health services**						
No problem						
Not a big problem						
Big problem						
**Distance to a health facility**						
No problem					1.00	1.00
Not a big problem					1.51 (1.20,1.89)	1.93 (1.51,2.46)
Big problem					1.62 (1.26,2.08)	1.07 (0.79,1.45)
**Postnatal check-up (PNC)**						
0–2 days				1.00	1.00	
3–41 days				1.45 (1.08,1.95)	0.97 (0.71,1.33)	
No PNC				1.06 (0.78,1.43)	0.63 (0.46,0.87)	
***Autonomy variables***						
**Power over household decision making**						
By Husband alone			1.00			
Alone/joint decision			1.21 (1.00,1.45)			
**Power over Earning**						
yes						
no						
**Attitudes to domestic violence**						
Yes						
No						
***Health Services***						
**Contraceptive use**						
Yes						
No						
**Intention to become pregnant**						
Now	1.00		1.00			
Later	1.36 (1.05,1.75)		1.16 (0.90,1.48)			
No more	1.26 (0.97,1.65)		1.34 (1.07,1.68)			

If 95% confidence intervals (CI) around AORs that lies between 1.00 indicate not statistically significant

Women with limited knowledge of delivery complications reported significantly higher odds of inadequate components ANC in the North (AOR = 1.18; 95%CI: 1.01 to 1.38) and East (AOR = 1.24; 95%CI: 1.08 to 1.41) regions. The odds of inadequate components of ANC in the Eastern and Central were significantly higher in women who intended not to become pregnant than in women who intended to become pregnant now by 26% and 34%, respectively. Older women (aged 30 plus) were significantly higher odds of inadequate components ANC in South and East regions. Place of delivery by inadequate components ANC varies lower odds in North region and higher odds in Central and North-eastern regions.

Women who had 2nd or 3rd birth rank infants and a short birth interval of less than or equal to two years reported higher odds of inadequate components of ANC in all regions except North and West regions. The odds of receiving inadequate components of ANC were significantly higher among women in Central (AOR = 1.62; 95%CI: 1.26 to 2.08) region who reported that distance to health services was a big problem. Participants who reported joint decision-making in the household were more likely to report inadequate components of ANC in the East region (AOR = 1.21; 95%CI: 1.00 to 1.45).

The odds of inadequate components of ANC in were significantly higher among women with no schooling in Jammu Kashmir. Women from poor households in Himachal Pradesh, Bihar, Uttar Pradesh, Nagaland, Manipur, Uttar Pradesh, and Madhya Pradesh states in India reported higher odds of inadequate components ANC than women from rich households. Women with limited knowledge of delivery complications reported significantly higher odds of inadequate components ANC in Himachal Pradesh, Punjab, Haryana, Delhi, Andhra Pradesh, Karnataka, Kerala, Tamil Nadu, Puducherry, Andaman Nicobar, Uttar Pradesh, Chhattisgarh, Madhya Pradesh, Sikkim, Nagaland, and Assam states in India. The receipt of inadequate components of ANC was significantly higher among women who never read magazines in Delhi, Ladakh, Karnataka, Telangana, Jharkhand, Maharashtra, Uttar Pradesh, Chhattisgarh, Arunachal Pradesh, Manipur and Mizoram states in India (see, Tables 1–6 in Supplementary Table 3 for details).

## Discussion

### Main findings

The components of ANC received by Indian women varied in the present study. Approximately seven percent (6.8%) of Indian women reported no ANC visit during pregnancy, and over three-quarters (80%) of Indian women reported inadequate receipts of ANC components, with varied subnational prevalence estimates. Several factors have emerged in this regional analysis that explain the inadequate receipt of ANC components and no ANC across India, and these associated factors vary by region, state, and Union territories; these factors are discussed below.

A recent study revealed that pregnant women in low and middle-income countries do not have access to all ANC components recommended by WHO [25]. This study found that the prevalence of inadequate receipt of ANC components and no ANC were lower (< 5.0%) in the Western and Eastern regions than the Indian national average. We reported that blood pressure check, weight check during pregnancy, and having blood and urine sample collected for investigation was relatively high (> 80%) in all regions of India. However, the prevalence of drugs for intestinal parasites during pregnancy was low in all six Indian regions. These findings are consistent with a district-level study conducted in India for ANC visits, Tetanus Toxoid vaccination (TT) and IFA, where the results showed that more emphasis needs to be given to the comprehensive provision of ANC components [10].

### Factors associated with inadequate receipt of ANC components

Our study found that residing in rural areas was associated with inadequate ANC components in the Central, Eastern regions and Lakshadweep states in India. This result was consistent with other studies conducted in developing countries [18–20]. A possible cause of inadequate receipt of ANC components in the rural Central, Eastern region and Lakshadweep states in India could be attributed to little or no access to media, low level of education, less availability of health facilities and skilled health personnel and cultural methods adopted in pregnancy [10]. This finding could also relate to the fact that major health centres and hospitals in India are located in urban areas, making rural pregnant women prefer receiving ANC components at home [26]. In addition, various programs, such as the free distribution of IFA supplements and drugs for intestinal parasites, should be integrated with ANC to improve the quality of ANC components provided at the rural level [19].

We found that women who reported limited knowledge about delivery complications had higher odds of inadequate receipt of ANC components including 16 states and union territories in India. These findings are consistent with a cross-sectional study conducted in Indonesia which found that women with inadequate receipt of ANC had higher odds of labour complications [22]. Similarly, a cross-sectional study conducted in Assem, India, found that women who received inadequate receipt of ANC were more likely to report delivery complications and post-delivery complications than those who received adequate ANC receipts [22]. These findings mirrored a recent study from India, which indicated that less than half of the pregnant women did not receive the minimum recommended 4 ANC visits [12]. A possible reason for the higher odds could be the distance to health facilities and limited knowledge about delivery and post-delivery complications [27]. Women may prefer not to deliver at the hospital due to the low-quality health facilities and distance to health facilities [28].

This study noted that women who delivered their babies at home reported higher odds of inadequate receipt of ANC than those who delivered their babies at the health facility in northern region and Gujarat state in West India; but the opposite finding was noted in central, north-eastern regions and Jammu Kashmir and Bihar state in India. The preference for home delivery among pregnant Indian women could be due to traditional cultural beliefs, the family’s financial ability to pay for health services, transport and access to the health facility, previous uncomplicated home deliveries, and fear of medical procedures, including obstetric examination [19, 25]. Women who had never listened to the radio and watched television reported mixed results across the six Indian regions. A cross-sectional study in India supported these findings and revealed a strong relationship between media exposure and at least eight ANC visits [29]. Also, insufficient access to media such as radio, magazines, and television could be linked to poor maternal awareness about ANC services [30]. Overcoming these barriers to inadequate receipt of ANC components by taking considerate actions at individual and community levels would enable the Indian government to achieve the SDG-3 of ensuring good health and promoting well-being.

Bruce et al. [19] women who reported later or had no intention to become pregnant had higher odds of inadequate receipt of ANC components in the North and Eastern regions. These findings agreed with a systematic review that examined the effect of ANC in developing countries which found that higher-order births were associated with increased odds of inadequate use of ANC [31].

In this study, we observed that women with no schooling and/or those from poorer households reported a higher likelihood of inadequate receipt of ANC components compared to their counterparts in almost all Indian regions, including 9 states. As previously suggested, women from low socioeconomic groups have limited knowledge about ANC visits and ANC components, delivery and post-delivery complications, and postnatal checkups or family planning [12, 27]. These findings are also consistent with other studies from low-and middle-income countries such as Nepal [32], Pakistan [33, 34] and Nigeria [18], which highlighted that low maternal education levels and poorest households are associated with inadequate receipt of ANC components. However, there is a need for the Indian government to educate women on how ways to improve their health outcomes, especially during pregnancy and by providing free ANC services, including ANC components for pregnant women from low socioeconomic groups, especially those rural women.

### Factors associated with no ANC

We found that women with no Schooling and/or those from poorer households were more likely to not utilise ANC services in many Indian regions, including 7 states. This finding is similar to other studies conducted in developing countries where low maternal education was one of the factors for no ANC visits [18, 33, 35]. A recent population-based study conducted in India in 2019 examining the enablers and barriers of ANC services revealed that maternal education was associated with several ANC visits [13].

Our study showed that women who delivered their babies at home were less likely not to use ANC services in the central, eastern, and western regions compared to their counterparts. However, the opposite result was noted in the northeastern region, where women who delivered their babies at the health facility were less likely not to use ANC services compared to women who delivered their babies at home. Part of the study finding supported research conducted in South Asia which showed that women who had more than three ANC visits reported a 7% higher probability of using a health facility for birthing compared to their counterparts who delivered at home with limited ANC visits [36]. Empowering Indian women through education would promote a better understanding of health messages, including enabling pregnant women to choose institutional delivery, better decision-making capacity in the household and knowledge about delivery and post-delivery complications [27].

The study showed that higher birth rank and long birth intervals reported higher odds of no ANC in all regions. These findings were supported by a cross-sectional study conducted in 12 East African countries which revealed that birth rank significantly affected ANC visits with decreased odds of birth rank among women who had four or more ANC visits [37]. A study conducted in regional Ethiopia found that birth interval was significantly higher among women who did not attend ANC [38]. The problem with distance to health facilities was related to limited ANC visits to southern India. This finding was consistent with research conducted in Nepal [32], Pakistan [33, 34] and Nigeria [25], which found that long distances to health facilities and access to ANC services were associated with No ANC visits.

Women who did not use a contraceptive and from socioeconomically disadvantaged groups reported higher odds of no ANC in all regions except the Southern regions. This is consistent with a community-based study conducted in Northeast Ethiopia which revealed that women from low socioeconomic status were less likely to use ANC [39], and a study that examined ways to improve the updated postpartum family planning in northern Tanzania found that counselling women during ANC did not improve contraceptive use [40].

### Study limitation and strengths

Limitations of this study were due to the cross-sectional design, which does not allow causal association between the study factors and outcome variables. Also, as ANC components were self-reported by pregnant women, recall bias, misclassification, and measurement of factors associated with no or inadequate receipt of ANC components. However, the major strengths of this study are that it utilised the most recent DHS, which provides a population-based, nationally representative sample and covers large sample sizes with a 98% response rate. The study has good internal validity as the questionnaire was validated by trained health personnel.

This study highlights the factors associated with no or inadequate receipt of ANC components at the regional level, states and union territories in India. Poor households, knowledge about delivery complications and post-delivery complications were significant factors associated with increased odds of receiving no or inadequate quality ANC in India, and these associated factors vary across all six regions. Home delivery reported higher odds of no ANC or inadequate quality ANC in all six regions except the East region. Women who had no postnatal checkups after delivery, low mother education level and a big problem with paying for health services reported higher odds of no ANC or inadequate quality ANC in all the various regions in India To improve women’s access to ANC services across all regions in India, it is prudent to consider the factors mentioned above to monitor and improve the maternal health programme in India, including media campaigns for creating awareness about ANC services, especially among pregnant women from lower socioeconomic groups.

## Supplementary Information

**Additional file 1: Supplementary Table 1.** Definition and categorisation of potential variables used in the study.

**Additional file 2: Table S1.** Factors associated with no ANC in North India states. **Table S2.** Factors associated with no ANC in South India states. **Table S3.** Factors associated with no ANC in East India states. **Table S4.** Factors associated with no ANC in West India states. **Table S5.** Factors associated with no ANC in Central India states. **Table S6.** Factors associated with no ANC in Northeast India states.

**Additional file 3: Table S1.** Factors associated with inadequate ANC in North India states. **Table S2.** Factors associated with inadequate ANC in South Indian states. **Table S3.** Factors associated with inadequate ANC in East India states. **Table S4.** Factors associated with inadequate ANC in West Indiana states. **Table S5.** Factors associated with inadequate ANC in Central India states. **Table S6.** Factors associated with inadequate ANC in Northeast Indian states.

**Additional file 4.** Prevalence of receipt of ANC by States and Union territories in India, 2019-21.

## Data Availability

The dataset analysed during the current study are available in the DHS Program repository, https://dhsprogram.com/.

## Abbreviations

ANC: Antenatal care
NDHS: Nigeria Demographic and Health Survey
WHO: World Health Organization
NFHS: National Family Health Survey
GBD: Global Burden of Disease
TT: Tetanus Toxoid vaccination
IFA: Iron-folic acid
SDGs: Sustainable Development Goals
LMIC: Low and middle-income countries
AOR: Adjusted Odds ratio
CI: Confidence interval
