# Supplementary material for: Factors associated with inadequate receipt of components and non-use of antenatal care services in India: a regional analysis
Source: BMC Public Health. 2023 Jan 3;23:6. doi: 10.1186/s12889-022-14812-3 (PMC9808929; doi:10.1186/s12889-022-14812-3)
Supplement: Supplementary file 1 — Additional file 1: Supplementary Table 1. Definition and categorisation of potential variables used in the study. [file 12889_2022_14812_MOESM1_ESM.pdf]

**Supplementary Table 1**. Definition and categorisation of potential variables used in the study

| Independent variables | Categorisation |
|---|---|
| **Community-level factor** | |
| Type of Residence | In the 2 following categories:(1=urban; 2=rural) |
| **Socio-economic factors** | |
| Maternal age at birth | In the 3 following categories: (1= 15-19 years; 2= 20-34 years; 3= 35-49 years) |
| Place of delivery | In the 2 following categories: (1= Home; 2= Health facility) |
| Household wealth Index | hv217 (the household wealth index factor score) constructed by DHS based on a selected set of household assets. In quintiles, 1= rich; 2& 3= middle; 4& 5= poor) |
| Maternal working status | In the 2 following categories: (1=not working; 2=working (for the past 12 months)) |
| Maternal education | In the 3 following categories: (1=Secondary or higher; 2=Primary; 3=No education) |
| Marital status | In the 2 following categories: (1= Currently married; 2=divorced/separated/widow) |
| Combined birth rank and birth Interval | (1= 2nd/3rd birth rank; 2=1st birth rank, more than 2 years interval; 3= 2nd/3rd birth rank, less than or equal to 2 years; 4= 4th birth rank, more than 2 years interval; 5= 4th birth rank, less than or equal to 2 years) |
| **Health Knowledge** | |
| Frequency of reading magazine or newspaper | In the 4 following categories: (1 =almost every day, 2 =At least once a week, 3 = Less than once a week, 4 = not at all) |
| Frequency of listening radio | In the 3 following categories: (1 =almost every day, 2 =At least once a week, 3 = Less than once a week, 4 = not at all) |
| Frequency of watching Television | In the 3 following categories: (1 =almost every day, 2 =At least once a week, 3 = Less than once a week, 4 = not at all) |
| Knowledge of delivery complications | In the 2 following categories: (1 =any complications; 2 = None) |
| Post-delivery complications knowledge | In the 2 following categories: (1 = Yes; 2 = None) |
| **Enabling factors** | |
| Permission to visit health services | In the 3 following categories:(1= No problem; 2= Not a big problem; 3= Big problem) |
| Money to pay for health services | In the 3 following categories:(1= No problem; 2= Not a big problem; 3= Big problem) |
| Not wanting to go alone to health care | In the 3 following categories:(1= No problem; 2= Not a big problem; 3= Big problem) |
| Distance to a health facility | In the 3 following categories:(1= No problem; 2= Not a big problem; 3= Big problem) |
| Postnatal check-up (PNC) | In the 3 following categories:(1= 0-2 days; 2= 3-41 days 3= No PNC) |
| *Women autonomy* | |
| Power over earning | In the 2 following categories:(1=Husband; 2= woman alone) |
| Power over household decision making | In the 2 following categories:(1=Husband; 2= woman alone) |
| Wife beaten for refusing sex | In the 2 following categories:(1=Yes; 2= No) |

| | |
|---|---|
| Attitudes to domestic violence | whether a husband was justified in beating his wife if she "goes out without telling him'; 'neglects the children';' refuses to have sex with him' 'argues with him'; or 'burns the food':(1=0 if she responded 'yes' (agreement) to any question, otherwise 2= 1 if she responded 'No (disagreement) to any question) |
| **Health Services** | |
| Antenatal care service attendant | In the 3 following categories:(1= Health practitioner; 2= No one, 3= Traditional) |
| Place received antenatal care services | In the 3 following categories:(1 =Government; 2= Private, 3= Home) |
| **Need factors** | |
| Contraceptive use | In the 2 following categories: (1 = Yes;   2 = No) |
| Intention to become pregnant | In the 3 following categories:(1 = Then; 2= Later, 3= No more) |
