# Supplementary material for: Factors associated with inadequate receipt of components and non-use of antenatal care services in India: a regional analysis
Source: BMC Public Health. 2023 Jan 3;23:6. doi: 10.1186/s12889-022-14812-3 (PMC9808929; doi:10.1186/s12889-022-14812-3)
Supplement: Supplementary file 2 — Additional file 2: Table S1. Factors associated with no ANC in North India states. Table S2. Factors associated with no ANC in South India states. Table S3. Factors associated with no ANC in East India states. Table S4. Factors associated with no ANC in West India states. Table S5. Factors associated with no ANC in Central India states. Table S6. Factors associated with no ANC in Northeast India states. [file 12889_2022_14812_MOESM2_ESM.pdf]

Table S1. Factors associated with no ANC in North India states.

| States | Jammu Kashmir | Himachal Pradesh | Punjab | Chandigarh | Uttarakhand | Haryana | Delhi | Rajasthan | Ladakh |
|---|---|---|---|---|---|---|---|---|---|
| **Variable** | AOR (95% CI) | AOR (95% CI) | AOR (95% CI) | AOR (95% CI) | AOR (95% CI) | AOR (95% CI) | AOR (95% CI) | AOR (95% CI) | AOR (95% CI) |
| **Maternal age at birth** | | | | | | | | | |
| <20 years | | | | | | | 1.00 | | |
| 20-29 years | | | | | | | 2.54 (0.93,6.95) | | |
| 30-39 years | | | | | | | 3.52 (1.28,9.65) | | |
| 40+ years | | | | | | | 38.54 (6.66,223.19) | | |
| **Place of delivery** | | | | | | | | | |
| Home | 1.00 | | | | | | | | |
| Health facility | 0.45 (0.31,0.66) | | | | | | | | |
| **Household wealth index** | | | | | | | | | |
| Rich | | | | | 1.00 | | | | |
| Middle | | | | | 0.31 (0.15,0.63) | | | | |
| Poor | | | | | 0.51 (0.26,1.02) | | | | |
| **Mother's education** | | | | | | | | | |
| Secondary | 1.00 | | | | | | | | |
| Primary | 0.78 (0.49,1.25) | | | | | | | | |
| No schooling | 0.69 (0.52,0.92) | | | | | | | | |
| **Frequency of watching Television** | | | | | | | | | |
| At least once a week | 1.00 | | | | | | | | |
| Less than once a week | 1.05 (0.76,1.46) | | | | | | | | |
| Not at all | 1.65 (1.21,2.26) | | | | | | | | |
| **Post-delivery complications knowledge** | | | | | | | | | |
| Yes | | | | | | | 1.00 | | |
| No | | | | | | | 3.43 (1.34,8.81) | | |
| **Postnatal check-up (PNC)** | | | | | | | | | |
| 0-2 days | 1.00 | | | | | | | | |

| | | | | | | | | |
|---|---|---|---|---|---|---|---|---|
| 3-41 days | 1.80 (1.04,3.11) | | | | | | | |
| No PNC | 2.25 (1.33,3.8) | | | | | | | |
| **Power over household decision making** | | | | | | | | |
| By Husband alone | | | | 1.00 | | | | |
| Alone/joint decision | | | | 3.90 (1.03,14.74) | | | | |
| **Intention to become pregnant.** | | | | | | | | |
| Now | | | | | 1.00 | | 1.00 | 1.00 |
| Later | | | | | 2.30 (0.88,6.04) | | 2.20 (1.41,3.44) | 1.44 (0.16,12.81) |
| No more | | | | | 3.74 (1.73,8.10) | | 1.63 (0.96,2.78) | 6.43 (1.05,39.55) |

If 95% confidence intervals (CI) around AORs that lies between 1.00 indicate not statistically significant

Table S2. Factors associated with no ANC in South India states.

| **States** | Andhra Pradesh | Karnataka | Lakshadweep | Kerala | Tamil Nadu | Puducherry | Andaman Nicobar | Telangana |
|---|---|---|---|---|---|---|---|---|
| **Variable** | AOR (95% CI) | AOR (95% CI) | AOR (95% CI) | AOR (95% CI) | AOR (95% CI) | AOR (95% CI) | AOR (95% CI) | AOR (95% CI) |
| **Type of residence** | | | | | | | | |
| Urban | | | 1.00 | | | | | |
| Rural | | | 0.18 (0.03,0.99) | | | | | |
| **Place of delivery** | | | | | | | | |
| Home | | 1.00 | | | | | | |
| Health facility | | 2.59 (1.20,5.60) | | | | | | |
| **Household wealth index** | | | | | | | | |
| Rich | 1.00 | 1.00 | | | | | | |
| Middle | 0.66 (0.43,1.02) | 0.79 (0.61,1.03) | | | | | | |
| Poor | 0.48 (0.29,0.78) | 0.61 (0.47,0.81) | | | | | | |
| **Frequency of reading magazines** | | | | | | | | |
| At least once a week | | | | 1.00 | | | | |
| Less than once a week | | | | 0.97 (0.65,1.46) | | | | |
| Not at all | | | | 1.72 (1.17,2.54) | | | | |
| **Frequency of watching Television** | | | | | | | | |
| At least once a week | | | | 1.00 | | | | 1.00 |

| | | | | |
|---|---|---|---|---|
| Less than once a week | | | 0.47 (0.26,0.85) | 0.54 (0.35,0.83) |
| Not at all | | | 1.24 (0.79,1.96) | 0.80 (0.49,1.33) |
| **Knowledge of delivery complications** | | | | |
| Yes | 1.00 | | | |
| No | 0.58 (0.35,0.97) | | | 1.00 |
| **Post-delivery complications knowledge** | | | | 0.61 (0.43,0.86) |
| Yes | | | | |
| No | | | | |
| **Distance to a health facility** | | | | |
| No problem | | | 1.00 | |
| Not a big problem | | | 0.49 (0.27,0.92) | |
| Big problem | | | 0.62 (0.10,3.78) | |
| **Postnatal check-up (PNC)** | | | | |
| 0-2 days | 1.00 | 1.00 | 1.00 | |
| 3-41 days | 0.40 (0.20,0.78) | 1.77 (1.04,3.03) | 3.55 (1.26,10.01) | |
| No PNC | 0.62 (0.33,1.16) | 1.72 (1.02,2.91) | 1.53 (0.53,4.47) | |

If 95% confidence intervals (CI) around AORs that lies between 1.00 indicate not statistically significant

Table S3. Factors associated with no ANC in East India states.

| **States** | Bihar | Odisha | Jharkhand | West Bengal |
|---|---|---|---|---|
| **Variable** | AOR (95% CI) | AOR (95% CI) | AOR (95% CI) | AOR (95% CI) |
| **Household wealth Index** | | | | |
| Rich | 1.00 | | 1.00 | |
| Middle | 0.46 (0.26,0.80) | | 0.55 (0.34,0.90) | |
| Poor | 0.29 (0.18,0.45) | | 0.37 (0.26,0.52) | |
| **Frequency of listening radio** | | | | |
| At least once a week | | | | 1.00 |
| Less than once a week | | | | 0.41 (0.19,0.85) |
| Not at all | | | | 0.40 (0.21,0.76) |
| **Power over household decision making** | | | | |
| By Husband alone | | | | 1.00 |

| | |
|---|---|
| Alone/joint decision | 1.48 (1.05,2.08) |

If 95% confidence intervals (CI) around AORs that lies between 1.00 indicate not statistically significant

Table S4. Factors associated with no ANC in West India states.

| States | Gujarat | Dadra & Nager Haveli and Daman and Diu | Maharashtra | Goa |
|---|---|---|---|---|
| **Variable** | AOR (95% CI) | AOR (95% CI) | AOR (95% CI) | AOR (95% CI) |
| **Frequency of listening to the radio** | | | | |
| At least once a week | | 1.00 | 1.00 | |
| Less than once a week | | 0.51 (0.27,0.94) | 0.51 (0.27,0.94) | |
| Not at all | | 0.46 (0.3,0.71) | 0.46 (0.30,0.71) | |
| **Frequency of reading magazines** | | | | |
| At least once a week | | | | |
| Less than once a week | | | | |
| Not at all | | | | |
| **Frequency of watching Television** | | | | |
| At least once a week | | 1.00 | 1.00 | |
| Less than once a week | | 0.77 (0.59,1.01) | 0.77 (0.59,1.01) | |
| Not at all | | 0.7 (0.53,0.93) | 0.70 (0.53,0.93) | |
| **Knowledge of delivery complications** | | | | |
| Yes | 1.00 | | | |
| No | 0.73 (0.59, 0.90) | | | |
| **Distance to a health facility** | | | | |
| No problem | | 1.00 | 1.00 | |
| Not a big problem | | 0.71 (0.54,0.94) | 0.71 (0.54,0.94) | |
| Big problem | | 0.87 (0.63,1.2) | 0.87 (0.63,1.20) | |
| **Postnatal check-up (PNC)** | | | | |
| 0-2 days | 1.00 | | | |
| 3-41 days | 1.41 (1.02, 1.97) | | | |
| No PNC | 1.05 (0.75, 1.47) | | | |

If 95% confidence intervals (CI) around AORs that lies between 1.00 indicate not statistically significant

Table S5. Factors associated with no ANC in Central India states.

| States | Uttar Pradesh | Chattisgarh | Madhya Pradesh |
|---|---|---|---|
| **Variable** | AOR (95% CI) | AOR (95% CI) | AOR (95% CI) |
| **Place of delivery** | | | |
| Home | 1.00 | | |
| Health facility | 1.97 (1.26,3.09) | | |
| **Household wealth Index** | | | |
| Rich | | 1.00 | |
| Middle | | 0.87 (0.58,1.32) | |
| Poor | | 0.66 (0.47,0.93) | |
| **Mother's education** | | | |
| Secondary | 1.00 | | |
| Primary | 0.66 (0.43,1.01) | | |
| No schooling | 0.46 (0.30,0.69) | | |
| **Frequency of reading magazines** | | | |
| At least once a week | | | 1.00 |
| Less than once a week | | | 0.54 (0.36,0.83) |
| Not at all | | | 0.44 (0.29,0.66) |
| **Postnatal check-up (PNC)** | | | |
| 0-2 days | 1.00 | | |
| 3-41 days | 0.89 (0.58,1.35) | | |
| No PNC | 0.57 (0.38,0.86) | | |

If 95% confidence intervals (CI) around AORs that lies between 1.00 indicate not statistically significant

Table S 6. Factors associated with no ANC in Northeast India states.

| States | Sikkim | Arunachal Pradesh | Nagaland | Manipur | Mizoram | Tripura | Meghalaya | Assam |
|---|---|---|---|---|---|---|---|---|
| **Variable** | AOR (95% CI) | AOR (95% CI) | AOR (95% CI) | AOR (95% CI) | AOR (95% CI) | AOR (95% CI) | AOR (95% CI) | AOR (95% CI) |
| **Type of residence** | | | | | | | | 1.00 |
| Urban | 1.00 | | | | | | | 0.56 (0.34,0.93) |
| Rural | 9.67 (2.40,38.91) | | | | | | | |
| **Household wealth Index** | | | | | | | | |

| | | | | | | |
|---|---|---|---|---|---|---|
| Rich | 1.00 | 1.00 | | | | |
| Middle | 1.08 (0.67,1.74) | 0.74 (0.51,1.09) | | | | |
| Poor | 2.75 (1.82,4.16) | 0.55 (0.41,0.74) | | | | |
| **Frequency of reading magazines** | | | | | | |
| At least once a week | | | | | 1.00 | |
| Less than once a week | | | | | 0.49 (0.23,1.06) | |
| Not at all | | | | | 0.33 (0.17,0.64) | |
| **Frequency of watching Television** | | | | | | |
| At least once a week | | | | | 1.00 | 1.00 |
| Less than once a week | | | | | 1.84 (1.02,3.31) | 0.71 (0.45,1.11) |
| Not at all | | | | | 1.50 (0.85,2.65) | 0.44 (0.27,0.72) |
| **Knowledge of delivery complications** | | | | | | |
| Yes | | | | 1.00 | | |
| No | | | | 1.49 (1.00,2.21) | | |
| **Distance to a health facility** | | | | | | |
| No problem | | | | 1.00 | | |
| Not a big problem | | | | 1.16 (0.69,1.94) | | |
| Big problem | | | | 2.13 (1.21,3.72) | | |
| **Contraceptive use** | | | | | | |
| Yes | | | 1.00 | | | |
| No | | | 2.46 (1.30,4.66) | | | |

If 95% confidence intervals (CI) around AORs that lies between 1.00 indicate not statistically significant
