# Supplementary material for: Factors associated with inadequate receipt of components and non-use of antenatal care services in India: a regional analysis
Source: BMC Public Health. 2023 Jan 3;23:6. doi: 10.1186/s12889-022-14812-3 (PMC9808929; doi:10.1186/s12889-022-14812-3)
Supplement: Supplementary file 3 — Additional file 3: Table S1. Factors associated with inadequate ANC in North India states. Table S2. Factors associated with inadequate ANC in South Indian states. Table S3. Factors associated with inadequate ANC in East India states. Table S4. Factors associated with inadequate ANC in West Indiana states. Table S5. Factors associated with inadequate ANC in Central India states. Table S6. Factors associated with inadequate ANC in Northeast Indian states. [file 12889_2022_14812_MOESM3_ESM.pdf]

Table S1. Factors associated with inadequate ANC in North India states.

States	Jammu Kashmir	Himachal Pradesh	Punjab	Chandigarh	Uttarakhand	Haryana	Delhi	Rajasthan	Ladakh
**Variable**	AOR (95% CI)	AOR (95% CI)	AOR (95% CI)	AOR (95% CI)	AOR (95% CI)	AOR (95% CI)	AOR (95% CI)	AOR (95% CI)	AOR (95% CI)
**Maternal age at birth**									
<20 years							1.00		
20-29 years							0.61 (0.36,1.02)		
30-39 years							0.61 (0.34,1.10)		
40+ years							0.07 (0.01,0.39)		
**Place of delivery**									
Home	1.00					1.00			
Health facility	1.65 (1.14,2.40)					0.26 (0.13,0.53)			
**Household wealth index**									
Rich	1.00	1.00							
Middle	1.01 (0.75,1.37)	1.11 (0.80,1.55)							
Poor	0.71 (0.54,0.94)	1.65 (1.07,2.53)							
**Mother's education**									
Secondary	1.00								
Primary	1.37 (0.89,2.12)								
No schooling	1.60 (1.25,2.05)								
**Combined Birth rank and Birth Interval**									
2nd/3rd >2years							1.00		
1st child							1.29 (1.01,1.65)		
2nd/3rd <=2years							1.14 (0.77,1.68)		
4th birth >2years							1.65 (0.57,4.80)		
4th birth <=2years							1.17 (0.36,3.79)		
**Frequency of listening to the radio**									
At least once a week							1.00		

Less than once a week							1.22 (0.78,1.90)		
Not at all							1.53 (1.02,2.29)		
**Frequency of reading magazines**									
At least once a week					1.00		1.00		1.00
Less than once a week					0.54 (0.31,0.95)		1.16 (0.80,1.68)		6.39 (2.18,18.74)
Not at all					0.66 (0.40,1.09)		1.69 (1.21,2.36)		2.16 (1.10,4.22)
**Frequency of watching Television**									
At least once a week	1.00		1.00		1.00			1.00	
Less than once a week	1.06 (0.80,1.41)		0.91 (0.71,1.17)		1.95 (1.26,3.04)			0.82 (0.67,0.99)	
Not at all	0.70 (0.52,0.93)		1.53 (1.10,2.13)		1.97 (1.16,3.34)			1.08 (0.89,1.30)	
**Knowledge of delivery complications**									
Yes		1.00	1.00			1.00	1.00		
No		5.86 (3.55,9.65)	2.35 (1.52,3.64)			5.00 (2.96,8.43)	4.48 (2.92,6.87)		
**Distance to a health facility**									
No problem			1.00	1.00			1.00		
Not a big problem			1.11 (0.88,1.40)	1.29 (0.20,8.21)			0.82 (0.62,1.08)		
Big problem			1.46 (1.10,1.94)	11.34 (1.28,100.8)			0.60 (0.40,0.90)		
**Postnatal check-up (PNC)**									
0-2 days	1.00					1.00	1.00		
3-41 days	0.50 (0.31,0.78)					1.01 (0.72,1.40)	1.04 (0.72,1.51)		
No PNC	0.47 (0.30,0.74)					1.73 (1.20,2.50)	1.67 (1.13,2.47)		
**Intention to become pregnant.**									
Now						1.00	1.00		
Later						1.09 (0.70,1.72)	2.06 (1.25,3.41)		
No more						2.26 (1.46,3.50)	1.87 (1.07,3.27)		

If 95% confidence intervals (CI) around AORs that lies between 1.00 indicate not statistically significant

Table S2. Factors associated with inadequate ANC in South Indian states.

**States**	Andhra Pradesh	Karnataka	Lakshadweep	Kerala	Tamil Nadu	Puducherry	Andaman Nicobar	Telangana
**Variable**	AOR (95% CI)	AOR (95% CI)	AOR (95% CI)	AOR (95% CI)	AOR (95% CI)	AOR (95% CI)	AOR (95% CI)	AOR (95% CI)
**Type of residence**								
Urban			1.00					
Rural			4.03 (1.05,15.38)					
**Household wealth Index**								
Rich	1.00	1.00						
Middle	1.04 (0.77,1.41)	1.08 (0.88,1.32)						
Poor	1.55 (1.10,2.18)	1.44 (1.15,1.80)						
**Frequency of reading magazines**								
At least once a week		1.00				1.00		1.00
Less than once a week		0.76 (0.60,0.97)				0.66 (0.34,1.29)		1.58 (1.16,2.16)
Not at all		1.14 (0.93,1.40)				0.45 (0.20,1.00)		1.56 (1.23,1.98)
**Frequency of watching Television**								
At least once a week		1.00		1.00		1.00		
Less than once a week		1.19 (0.92,1.54)		2.02 (1.35,3.04)		1.42 (0.52,3.90)		
Not at all		1.44 (1.11,1.88)		0.62 (0.43,0.90)		3.25 (1.42,7.47)		
**Knowledge of delivery complications**								
Yes	1.00	1.00		1.00	1.00	1.00	1.00	
No	5.64 (3.46,9.20)	4.28 (3.27,5.61)		2.47 (1.78,3.43)	12.06 (8.88,16.36)	9.39 (2.38,37.11)	42.23 (9.46,188.45)	
**Post-delivery complications knowledge**								
Yes	1.00	1.00			1.00			
No	4.09 (2.19,7.64)	0.41 (0.20,0.85)			2.13 (1.31,3.46)			
**Distance to a health facility**								
No problem					1.00			
Not a big problem					1.23 (0.95,1.6)			
Big problem					1.27 (1.03,1.58)			
**Postnatal check-up (PNC)**								
0-2 days				1.00	1.00			1.00
3-41 days				0.49 (0.26,0.95)	0.83 (0.65,1.07)			1.50 (1.04,2.17)

No PNC			0.93 (0.48,1.80)	1.40 (1.07,1.84)			1.65 (1.21,2.25)
**Intention to become pregnant.**							
Now	1.00		1.00	1.00			1.00
Later	2.30 (1.04,5.07)		2.42 (1.34,4.37)	3.76 (2.01,7.05)			1.00 (0.62,1.62)
No more	2.05 (1.22,3.43)		1.38 (0.60,3.19)	3.34 (1.89,5.90)			0.50 (0.33,0.78)

If 95% confidence intervals (CI) around AORs that lies between 1.00 indicate not statistically significant

Table S3. Factors associated with inadequate ANC in East India states.

**States**	Bihar	Odisha	Jharkhand	West Bengal
**Variable**	AOR (95% CI)	AOR (95% CI)	AOR (95% CI)	AOR (95% CI)
**Maternal age at birth**				
<20 years				1.00
20-29 years				0.85 (0.70,1.04)
30-39 years				0.62 (0.45,0.84)
40+ years				0.46 (0.12,1.72)
**Place of delivery**				
Home	1.00	1.00	1.00	1.00
Health facility	1.31 (1.10,1.55)	0.68 (0.54,0.85)	0.55 (0.38,0.79)	0.58 (0.39,0.87)
**Household wealth Index**				
Rich	1.00		1.00	
Middle	1.90 (1.37,2.63)		1.43 (0.98,2.08)	
Poor	2.00 (1.52,2.63)		1.80 (1.31,2.49)	
**Mother's education**				
Secondary		1.00		1.00
Primary		0.82 (0.69,0.98)		1.36 (1.08,1.73)
No schooling		0.86 (0.72,1.02)		1.25 (0.94,1.65)
**Frequency of reading magazines**				
At least once a week			1.00	
Less than once a week			1.16 (0.81,1.67)	
Not at all			2.12 (1.45,3.10)	
**Frequency of watching Television**				1.00

At least once a week				1.14 (0.91,1.43)
Less than once a week				1.40 (1.14,1.71)
Not at all				
**Knowledge of delivery complications**				
Yes	1.00		1.00	
No	1.39 (1.16,1.67)		6.44 (3.61,11.49)	
**Distance to a health facility**				
No problem	1.00			
Not a big problem	0.92 (0.75,1.12)			
Big problem	0.80 (0.65,0.98)			
**Postnatal check-up (PNC)**				
0-2 days		1.00		
3-41 days		0.90 (0.72,1.11)		
No PNC		2.12 (1.66,2.71)		
**Power over household decision making**				
By Husband alone				1.00
Alone/joint decision				0.77 (0.59,0.99)
**Contraceptive use**				
Yes				
No	0.82 (0.70,0.95)			
**Intention to become pregnant.**				
Now		1.00	1.00	
Later		1.48 (1.12,1.97)	2.55 (1.18,5.52)	
No more		1.43 (1.01,2.02)	0.75 (0.37,1.50)	

If 95% confidence intervals (CI) around AORs that lies between 1.00 indicate not statistically significant

Table S4. Factors associated with inadequate ANC in West Indiana states.

**States**	Gujarat	Dadra & Nager Haveli and Daman and Diu	Maharashtra	Goa
**Variable**	AOR (95% CI)	AOR (95% CI)	AOR (95% CI)	AOR (95% CI)
**Maternal age at birth**				

<20 years	1.00			
20-29 years	1.10 (0.82,1.47)			
30-39 years	1.16 (0.84,1.61)			
40+ years	2.63 (1.00,6.92)			
**Place of delivery**				
Home	1.00			
Health facility	0.60 (0.39,0.94)			
**Frequency of listening radio**				
At least once a week				
Less than once a week			2.09 (1.36,3.19)	
Not at all			2.17 (1.53,3.06)	
**Frequency of reading magazines**				
At least once a week				
Less than once a week		4.09 (1.33,12.61)	1.02 (0.81,1.29)	
Not at all		7.11 (2.66,19.04)	1.52 (1.21,1.90)	
**Knowledge of delivery complications**				
Yes				
No			1.88 (1.52,2.32)	
**Distance to a health facility**				
No problem				
Not a big problem			1.29 (1.05,1.58)	
Big problem			1.28 (1.03,1.59)	
**Postnatal check-up (PNC)**				
0-2 days				
3-41 days		0.10 (0.02,0.47)		2.94 (0.73, 11.79)
No PNC		0.20 (0.04,1.00)		6.14 (1.58, 23.85)
**Intention to become pregnant.**				
Now	1.00			
Later	2.40 (1.33,4.32)			
No more	1.76 (1.02,3.06)			

If 95% confidence intervals (CI) around AORs that lies between 1.00 indicate not statistically significant

Table S5. Factors associated with inadequate ANC in Central India states.

States	Uttar Pradesh	Chattisgarh	Madhya Pradesh
**Variable**	AOR (95% CI)	AOR (95% CI)	AOR (95% CI)
**Place of delivery**			
Home	1.00	1.00	1.00
Health facility	0.62 (0.50,0.77)	0.63 (0.46,0.88)	0.66 (0.47,0.92)
**Household wealth index**			
Rich	1.00		1.00
Middle	1.07 (0.90,1.27)		1.26 (0.95,1.67)
Poor	1.18 (1.00,1.39)		1.45 (1.18,1.78)
**Mother's education**			
Secondary	1.00		
Primary	1.23 (1.01,1.51)		
No schooling	1.14 (0.97,1.34)		
**Frequency of reading magazines**			
At least once a week	1.00	1.00	
Less than once a week	0.96 (0.73,1.26)	0.91 (0.64,1.31)	
Not at all	1.39 (1.07,1.80)	1.25 (0.92,1.70)	
**Knowledge of delivery complications**			
Yes	1.00	1.00	1.00
No	8.32 (6.08,11.4)	11.15 (4.36,28.53)	7.16 (4.78,10.73)
**Post-delivery complications knowledge**			
Yes	1.00	1.00	
No	0.53 (0.34,0.83)	1.91 (1.14,3.20)	
**Postnatal check-up (PNC)**			
0-2 days	1.00	1.00	1.00
3-41 days	0.93 (0.74,1.17)	0.66 (0.49,0.90)	0.82 (0.61,1.11)
No PNC	1.31 (1.03,1.65)	1.00 (0.72,1.38)	1.35 (1.00,1.84)
**Contraceptive use**			
Yes	1.00		
No	1.15 (1.00,1.31)		

**Intention to become pregnant.**	
Now	1.00
Later	1.66 (1.09,2.53)
No more	2.09 (1.45,3.02)

If 95% confidence intervals (CI) around AORs that lies between 1.00 indicate not statistically significant

Table S6. Factors associated with inadequate ANC in Northeast Indian states.

**States**	Sikkim	Arunachal Pradesh	Nagaland	Manipur	Mizoram	Tripura	Meghalaya	Assam
**Variable**	AOR (95% CI)	AOR (95% CI)	AOR (95% CI)	AOR (95% CI)	AOR (95% CI)	AOR (95% CI)	AOR (95% CI)	AOR (95% CI)
**Place of delivery**								
Home		1.00		1.00		1.00		
Health facility		3.73 (2.97,4.68)		1.50 (1.09,2.08)		0.39 (0.16,0.95)		
**Household wealth index**								
Rich			1.00	1.00				
Middle			7.46 (2.42,22.97)	1.13 (0.80,1.59)				
Poor			10.62 (2.11,53.33)	1.70 (1.23,2.35)				
**Frequency of listening to radio**								
At least once a week							1.00	
Less than once a week							1.58 (0.79,3.15)	
Not at all							1.97 (1.09,3.58)	
**Frequency of reading magazines**								
At least once a week		1.00		1.00	1.00			
Less than once a week		0.93 (0.56,1.55)		1.14 (0.77,1.67)	1.32 (0.69,2.56)			
Not at all		1.80 (1.15,2.80)		1.55 (1.07,2.25)	4.68 (1.84,11.87)			
**Frequency of watching Television**								
At least once a week	1.00							
Less than once a week	0.48 (0.25,0.93)							
Not at all	0.82 (0.34,1.95)							
**Knowledge of delivery complications**								
Yes	1.00	1.00	1.00				1.00	1.00
No	5.01 (2.1,11.94)	0.75 (0.60,0.94)	10.55 (1.48,75.24)				0.28 (0.21,0.38)	3.81 (2.25,6.43)

**Distance to a health facility**				
No problem	1.00	1.00	1.00	1.00
Not a big problem	0.88 (0.63,1.21)	1.36 (1.02,1.81)	4.21 (1.36,13.05)	1.27 (0.93,1.73)
Big problem	0.72 (0.53,0.99)	1.02 (0.60,1.74)	0.30 (0.10,0.89)	1.55 (1.11,2.17)
**Intention to become pregnant.**				
Now				
Later			1.00	
No more			0.37 (0.16,0.88)	

If 95% confidence intervals (CI) around AORs that lies between 1.00 indicate not statistically significant
