# Supplementary figures and images for: Factors associated with inadequate receipt of components and non-use of antenatal care services in India: a regional analysis
Source: BMC Public Health. 2023 Jan 3;23:6. doi: 10.1186/s12889-022-14812-3 (PMC9808929; doi:10.1186/s12889-022-14812-3)

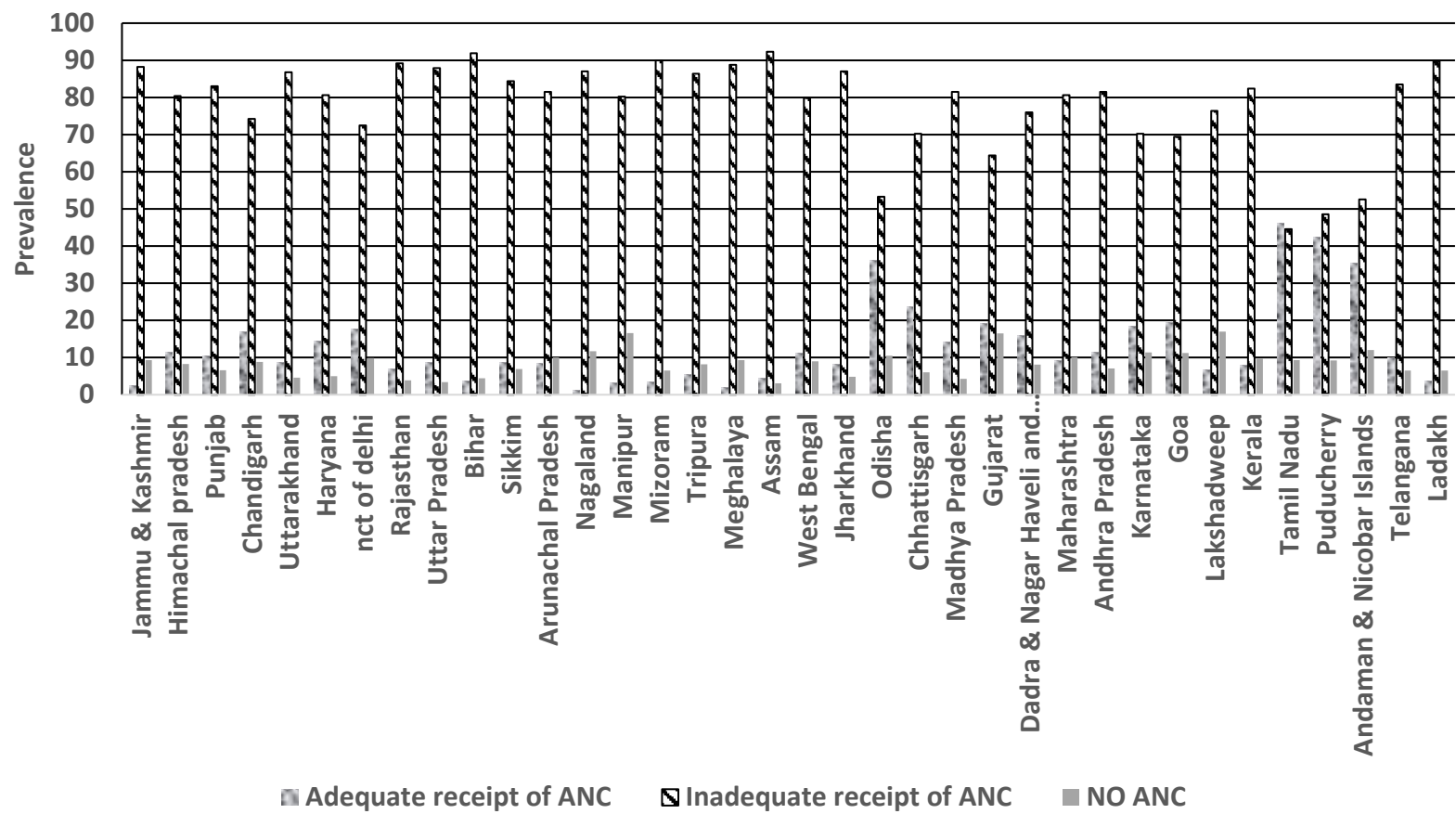

Prevalence of receipt of ANC by States and Union territories in India, 2019-21

Supplement: Supplementary file 4 — Additional file 4. Prevalence of receipt of ANC by States and Union territories in India, 2019-21. [file 12889_2022_14812_MOESM4_ESM.pdf]
